# Relationship between cathepsin K and extracellular matrix dynamics: a comprehensive review

**DOI:** 10.3389/fonc.2026.1761157

**Published:** 2026-03-20

**Authors:** Guodong Zang, Tingting Wang, Hao Tian, Yijie Wang, Dengru Jia, Rui Fan

**Affiliations:** 1 Respiratory and Critical Care Medicine Department, Affiliated Hospital of Shandong University of Traditional Chinese Medicine, Jinan, Shandong, China; 2 Department of Hepatology, Shandong Provincial Third Hospital, Shandong University, Jinan Shandong, China; 3 Respiratory and Critical Care Medicine Department, Linyi Hospital of Traditional Chinese Medicine, Linyi, Shandong, China; 4 The First Clinical Medical College, Shandong University of Traditional Chinese Medicine, Jinan, Shandong, China; 5 Graduate School, Shandong First Medical University, Jinan, Shandong, China; 6 Respiratory and Critical Care Medicine Department, Provincial Hospital Affiliated to Shandong First Medical University, Jinan, Shandong, China

**Keywords:** cancer, cathepsin K, CTSK, extracellular matrix (ECM), epithelial to mesenchymal transformation (EMT)

## Abstract

**Objective:**

To systematically review the pleiotropic roles of Cathepsin K (CTSK) beyond classical bone resorption, elucidating its critical function in regulating extracellular matrix (ECM) dynamics and epithelial-mesenchymal transition (EMT) across diverse pathological systems.

**Methods:**

A comprehensive analysis was conducted to synthesize the molecular architecture and signaling networks of CTSK, including the RANKL-RANK and NF-κB pathways. The review stratifies mechanistic and clinical evidence across five major domains: malignant tumors, cardiovascular diseases, pulmonary disorders, orthopedic conditions, and metabolic diseases, while evaluating the development and risks of CTSK inhibitors.

**Results:**

CTSK acts as a versatile protease in ECM remodeling. In oncology, it facilitates metastasis in breast, gastric, and prostate cancers by degrading matrix barriers and activating EMT. In cardiovascular and pulmonary pathologies, CTSK exhibits a dualistic nature: it promotes atherosclerotic plaque instability and fibrosis progression but is protective in ischemic vascular remodeling. Furthermore, in metabolic disorders like T2DM and obesity, upregulated CTSK drives pathological collagen degradation, compromising tissue integrity. In orthopedic diseases, it is a key effector molecule responsible for bone matrix degradation and impaired tissue repair. Therapies targeting CTSK (such as inhibitors) show promise but raise safety concerns including off-target effects and increased stroke risk.

**Conclusion:**

CTSK is a central hub integrating upstream signals to regulate systemic ECM homeostasis, making it a promising therapeutic target. Future therapeutic strategies should focus on developing highly selective inhibitors to achieve precise regulation and balance efficacy with safety.

## 1 Introduction

Cathepsin K (CTSK) is a lysosomal cysteine protease initially identified as being highly expressed in mature osteoclasts. It efficiently cleaves key matrix components such as type I collagen in acidic environments, driving rapid degradation and remodeling of the bone matrix (1, 2). Cathepsin K is distinguished among mammalian proteases for its capacity to hydrolyze natural collagen helices, rendering it a fundamental hydrolytic enzyme in bone resorption (3). The CTSK gene, which encoded Cathepsin K protein, is located in the 1q21 region, encoding a precursor protein of approximately 38kDa, which is subsequently processed into the active enzyme within the lysosome (4). Orthopedic diseases resulting from genetic deletion or inactivation (such as achondroplasia) confirm the irreplaceable role of CTSK in maintaining bone matrix homeostasis (5). Recent studies have shown that the function of CTSK extends far beyond bone metabolism itself. In addition to osteoclasts, CTSK is also expressed in fibroblasts, macrophages, and various tumor cells, participating in multiple biological processes including extracellular matrix (ECM) dynamics (6, 7).

In 1995, Shi et al. (8) first reported the molecular cloning and characterization of a novel human cysteine protease, designated cathepsin O (Currently known as cathepsin K/CTSK), which shares 94% identity with rabbit gene OC2 and over 50% identity with human cathepsins S (CTSS) (9) and cathepsins L (CTSL) (10), and demonstrated its potent endoproteolytic activity against fibrinogen in an acidic environment (8, 11). This study indicated that the expression of cathepsin O/K is selectively elevated during the late-stage maturation of human monocyte-derived macrophages, implying its possible function in specialized ECM degradation (8). Following the initial identification of the novel cysteine protease (OC-2/cathepsin K) in rabbit osteoclasts, its human and murine homologs were subsequently cloned, revealing a highly conserved molecule across species (12, 13). The specific expression pattern of CTSK in bone and cartilage indicates that it is a key mediator influencing ECM dynamics.

The cathepsin K, encoded by the CTSK gene, plays a variety of biological functions and constitutes a complex regulatory network by coordinating key mechanisms such as ECM remodeling and epithelial-mesenchymal transition (EMT). At the transcriptional level, cathepsin K is a multifunctional protease whose expression is regulated by signaling pathways such as RANKL, TNF-α, estrogen, c-Jun, and MITF (14). At the epigenetic level, cathepsin K can enter the nucleus of osteoblasts and cleave histone H3K27me3, thereby regulating the expression profile of osteogenic-related genes and affecting key transcription factors such as CEBPA and NFATC1 (15). Its collagenase activity is significantly enhanced after forming a complex with glycosaminoglycans (such as C4-S), and negatively charged polymers can selectively inhibit this complex, thus achieving specific regulation of collagen degradation (16).

Since cathepsin K was first identified in bone tissue, it is associated with several pathological processes in extraskeletal tissues. For instance, it is highly expressed in atherosclerotic plaques, where it promotes vascular smooth muscle cell proliferation and intimal hyperplasia (17). In the context of rheumatoid arthritis and periodontitis, it participates in the immune-inflammatory responses of dendritic cells, macrophages, and T cells by regulating signaling axes such as NF-κB, TLR, and RANKL/RANK/OPG (7). Within gastrointestinal epithelial cells and dermal fibroblasts, it mediates the lysosomal degradation of collagen; its expression is upregulated by IL-1g and high cell density but inhibited by TGF-bit Additionally, this enzyme promotes the fibrotic process following muscle injury (7). In various tumors, including breast cancer, it facilitates tumor metastasis through mechanisms such as degrading the extracellular matrix and activating PAR-3/4 receptors (18). Cathepsin K is also secreted by thyroid cells to proteolyze thyroglobulin at neutral pH, suggesting a role in hormone liberation (19). Furthermore, cathepsin K exhibits a dual regulatory role in diseases such as pulmonary fibrosis.

**Figure 1:**
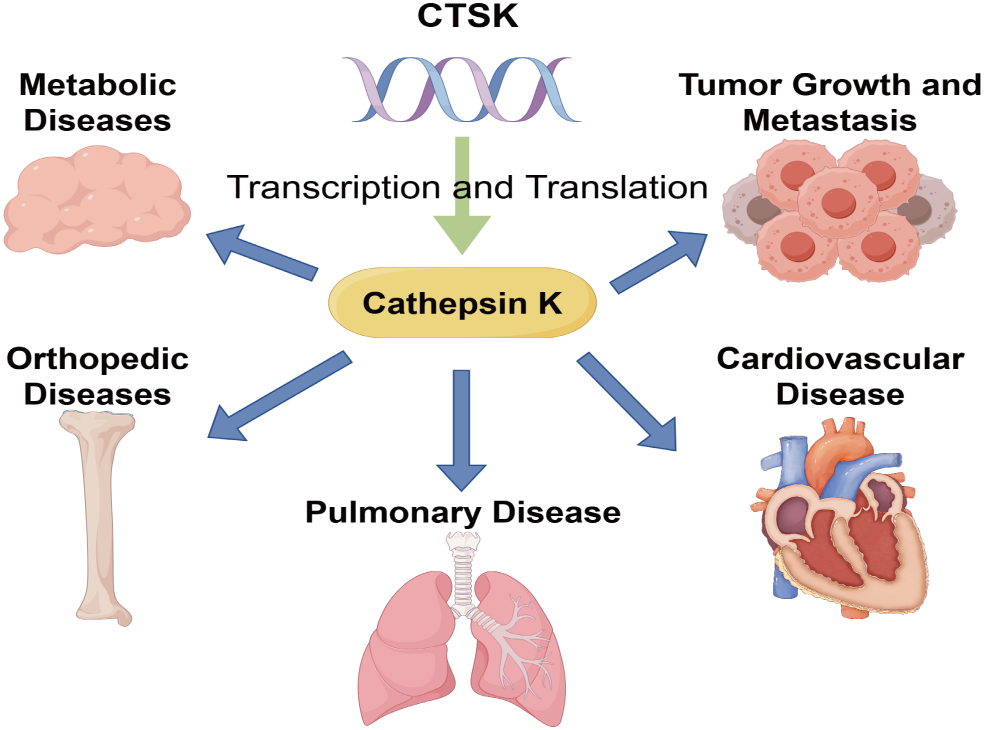
Flowchart of the systematic review process on the relationship between cathepsin K (CTSK) and ECM dynamics.

The article initiates with an examination of the molecular structure of CTSK, investigating its influence on ECM remodeling and EMT pathways in diverse clinical conditions, including malignant tumors, pulmonary disorders, and cardiovascular diseases, Orthopedic diseases and metabolic diseases, while summarizing CTSK’s effect on ECM dynamics. It will be introduced in further detail below. The research process is shown in **Figure 1**.

## 2 Molecular architecture and functions of cathepsin K

Since its first identification in osteoclasts three decades ago, CTSK has been thoroughly investigated. In 1995, Drake et al. first discovered that CTSK was highly expressed in osteoclasts by random sequencing of human osteoclast cDNA libraries, while its transcripts were almost undetectable in other tissues (1). Subsequently, Rood et al. completed the CTSK genome structure analysis in 1997, confirming that it is located in chromosome 1 (20). Functional experiments at the same time showed that the polar distribution of CTSK in osteoclasts highly overlapped with the contact area on the bone surface, suggesting its role in bone matrix degradation (1). At a later stage, pycnodysostosis, a rare hereditary skeletal disease caused by CTSK deficiency, was reported, further confirming the biological role of CTSK in ECM dynamics (21).

**Figure 2:**
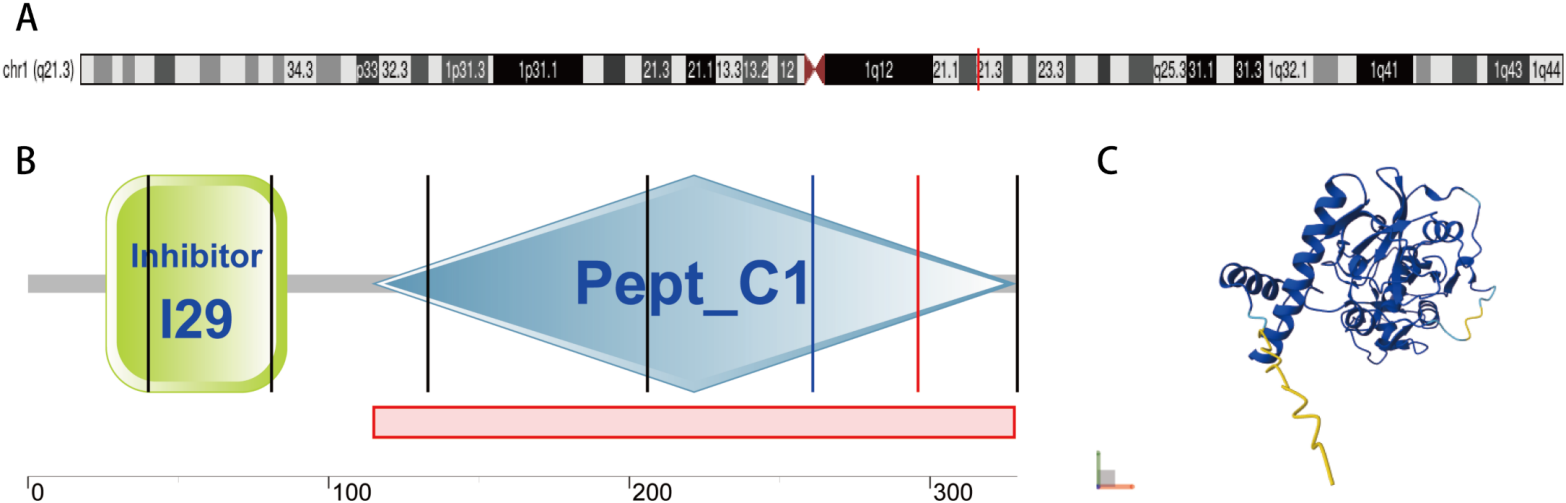
Domains within Homo sapiens protein CTSK (CATK_HUMAN, P43235). **(A)** Location of CTSK on chromosome 1; **(B)** Schematic representation of CTSK (22): Green section represents a peptidase inhibitor domain, which belongs to MEROPS peptidase inhibitor family I29. Gray section represents the papain C-terminal of a group of proteins that belong to the cysteine peptidase family C1, sub-family C1A (papain family, clan CA). **(C)** AlphaFold Model of CTSK (AF-P43235-F1-v6) (23), model confidence: dark blue: very high (pLDDT >90); light blue: confident (90 > pLDDT >70); yellow: low (70 > pLDDT >50); orange: very low (pLDDT <50), AlphaFold produces a per-residue confidence score (pLDDT) between 0 and 100. Some regions below 50 pLDDT may be unstructured in isolation.

The CTSK consists of 8 exons and 7 introns, covering roughly 9–12 kb of genomic DNA. The promoter region is devoid of canonical TATA and CAAT boxes but includes potential regulatory elements like AP1 sites, which may facilitate its selective expression in osteoclasts and macrophages. Transcription commences from a singular start point situated 49–169 bp upstream of the translation starting codon, contingent upon the study (21). CTSK is located at chromosome 1q21.3, with its genomic coordinates defined as 1:150,796,208-150,808,260 (Genome Reference Consortium 38, GRCh38), as shown in **Figure 2A**. This mapping positions CTSK within 150 kb of the evolutionarily related cathepsin S gene (CTSS), suggesting a possible gene cluster arrangement. This genomic characterization elucidates the structural and regulatory foundations of CTSK function and enables mutation analysis in various illnesses (20, 24).

Cathepsin K is a papain−like lysosomal cysteine protease synthesized as a 329−residue pre−proenzyme that contains a 15−residue signal peptide, a -99−residue propeptide, and a 215−residue mature catalytic domain; the active site is formed by the canonical Cys−His−Asn triad (24). X−ray crystallography reveals a conserved papain core but a distinctive positively charged surface and two auxiliary exosites that together create a wide, shallow substrate−binding pocket, enabling efficient cleavage of the triple−helical region of type I collagen (21, 25). The protein structure of cathepsin K is shown in **Figures 2B, C**, and **Table 1**. *In vivo*, cathepsin K, which is highly expressed in osteoclasts and serves as the primary enzyme responsible for collagen degradation in bone resorption, is critical for skeletal homeostasis; accordingly, its loss-of-function mutations are causative of various orthopedic disorders (26, 27). Indeed, as previously noted, cathepsin K serves as a pervasive regulator in other pathological situations beyond bone remodeling. Although cathepsin K is a major therapeutic target for osteoporosis (OP) and other conditions involving extracellular matrix remodeling and EMT due to its potent collagenolytic activity (28), the potential risks associated with its inhibition warrant careful consideration, which are discussed in section 8.

**Table 1:** Domains within Homo sapiens protein cathepsin K.

Classification	Feature	Start	End	E-value
Confidently predicted domains, repeats, motifs and features	Inhibitor_I29	26	86	5.53e-22
	Pept_C1	115	328	2.26e-116
Outlier homologues and homologues of known structure	PDB:5Z5O|A	8	329	0.00e+00
	Blast: Pept_C1	115	328	6.60e-158
	SCOP:8098809	115	329	1.61e-161
Features NOT shown in the diagram	Pfam: Inhibitor_I29	26	86	7.30e-14
	Pfam: Peptidase_C1	115	328	1.50e-85
	Pfam: Peptidase_C1_2	261	316	8.80e-08

## 3 Fundamental signaling pathways and regulatory mechanisms of CTSK

**Figure 3:**
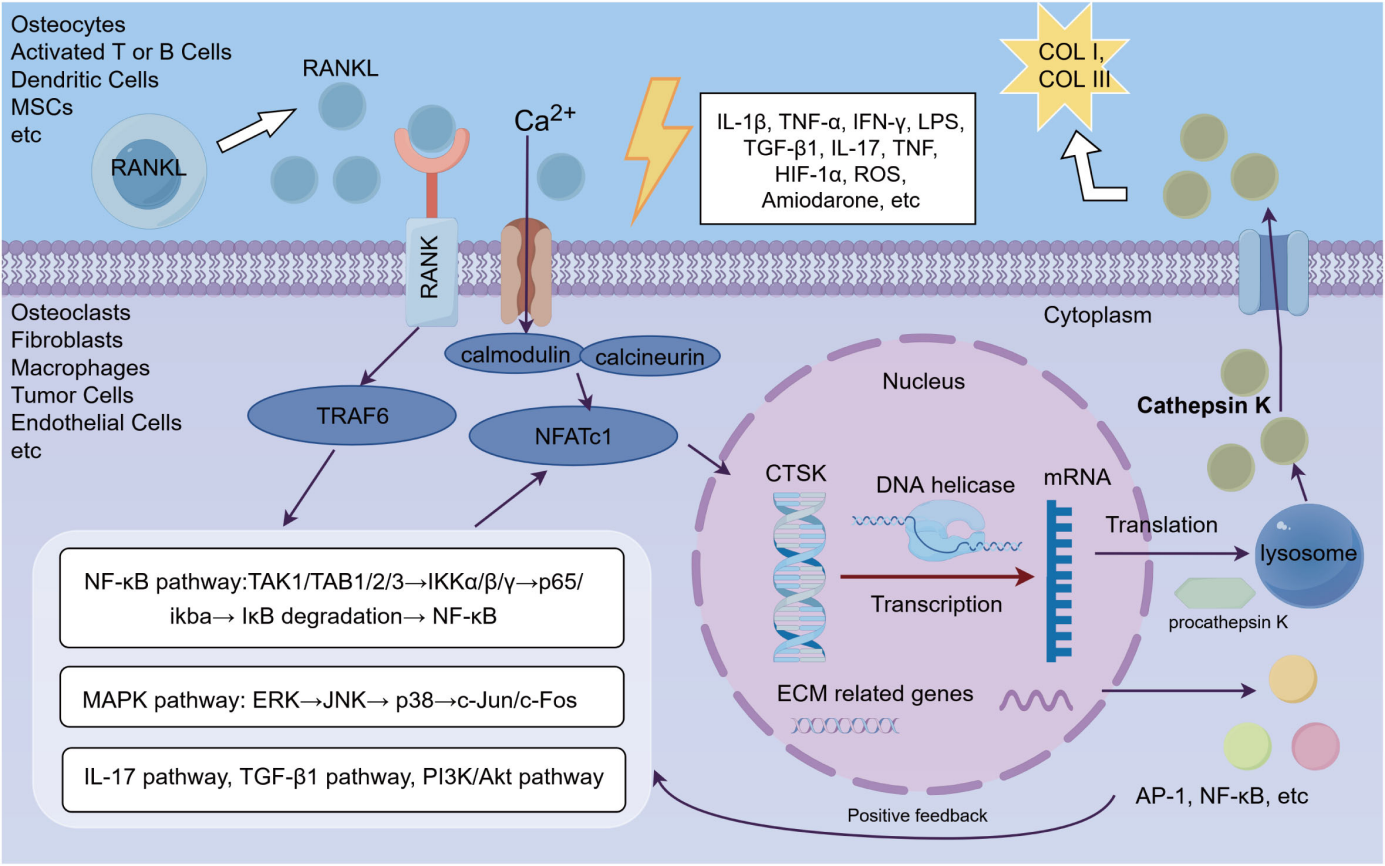
CTSK signal transduction mechanism. Mechanisms of CTSK. CTSK, a key effector downstream of the RANKL–RANK signaling pathway, mediates the degradation of ECM components. Its expression extends beyond osteoclasts to diverse cell types, where it contributes to bone resorption, cancer progression, and immune regulation.

CTSK is a key effector molecule downstream of the RANKL-RANK signaling pathway, primarily responsible for degrading ECM components, especially COLI and COLIII. CTSK expression is meticulously controlled by the RANKL-RANK signaling pathway, exhibiting elevated levels during osteoclast development and activation. The RANKL-RANK interaction initiates a downstream signaling cascade, including the recruitment of tumor necrosis factor receptor-associated factor 6 (TRAF6) (29). Upon activation, TRAF6 subsequently activates the NF-iv and MAPK signaling pathways. The NF-κB pathway promotes the degradation of IκB inhibitory proteins through phosphorylation, releasing NF-κB into the nucleus to initiate transcription programs of osteoclast differentiation-specific genes (30). The MAPK pathway, encompassing extracellular signal-regulated kinase (ERK), c-Jun N-terminal kinase (JNK), and p38 kinase, also plays a crucial role in this process (30, 31). Additionally, RANKL activates nuclear factor of activated T cells, factor c1 (NFATc1) through the calcium/calmodulin/calcineurin signaling pathway. NFATc1, the primary regulator of osteoclast differentiation, translocates to the nucleus upon activation, where it collaborates with NF-ha and additional proteins (such as AP-1) to establish a positive feedback loop that enhances CTSK synthesis in (32). Following enhanced CTSK expression, its corresponding protein is primarily secreted via the lysosomal exocytosis mechanism. In osteoclasts, secreted CTSK degrades the ECM and other matrix proteins, playing a crucial role in bone resorption (33). Although CTSK was initially thought to be primarily expressed in osteoclasts, subsequent studies revealed its expression in various other cell types, including tumor cells, macrophages, T cells, fibroblasts, adipocytes, and certain mesenchymal stem cells (Refer to the information below). CTSK in these cells may be synthesized and released through distinct mechanisms and pathways, playing roles in multiple pathophysiological processes such as cancer progression, immune regulation, and inflammation (**Figure 3**).

CTSK are primarily localized within the endosomal system, released from lysosomes into the extracellular space, and maintain their proteolytic activity through a series of complex mechanisms (34). CTSK is initially synthesized as an inactive precursor enzyme (procathepsin K), which requires autocatalytic cleavage in the acidic environment of lysosomes or secretory vesicles to remove the N-terminal propeptide and form the active mature enzyme. This process typically occurs within an acidic microenvironment at a pH of approximately 4.5, ensuring the enzyme’s activation and functional expression under specific physiological conditions (34, 35). Additionally, cells induce conformational changes in CTSK and coordinate with matrix components like Glycosaminoglycans (GAGs) to exert allosteric regulation (36). Together, these mechanisms form a sophisticated network that maintains and regulates CTSK’s proteolytic activity outside lysosomes, thereby sustaining CTSK homeostasis.

## 4 Relationship between cathepsin K and ECM dynamics in tumor growth and metastasis

Cancer remains a leading cause of morbidity and mortality in the United States, with an estimated 2.0 million new cases and over 600,000 deaths projected for 2025 (37). Although overall cancer mortality has declined steadily over recent decades, the disease continues to present a substantial public health challenge—particularly in light of the concerning upward trend in incidence among younger and middle-aged women, which reflects a dynamic and increasingly complex epidemiological landscape (37). Notably, the following cancer types exhibit significantly high incidence rates: lung cancer, colorectal cancer, pancreatic ductal adenocarcinoma, gastric cancer (GC), breast cancer, head and neck squamous cell carcinoma, prostate cancer, and hepatocellular carcinoma (37, 38). Numerous independent studies have shown that CTSK, as an oncogene, affects tumor cell proliferation, invasion, and distant metastasis by regulating ECM dynamics in various cancer types, including breast cancer, GC, prostate cancer, etc, altering matrix stiffness and accessibility of bioactive sites (4). CTSK-mediated dynamic remodeling of the ECM not only facilitates tumor cell invasion by creating permissive migratory channels, but also promotes aggressive tumor growth through the release of multiple growth-promoting factors, including TGF-β1 (39), FGF (40), VEGF (41), IGF1R (42), and EGFR (43), which collectively enhance proliferative signaling pathways. At the same time, the reduction in matrix stiffness and the rearrangement of adhesion sites enhance the migration ability of cancer cells, promoting vascular endothelial invasion and distant metastasis (44). **Table 2** illustrates the function of CTSK in tumor growth and metastasis.

**Table 2:** The function of CTSK in tumor growth and metastasis.

Gene	Disease	Expression	Substrates/target	Study type	Sample	References
C T S K	Breast cancer	Upregulated	–	Comparative cross-sectional study	Human breast tumor tissue	(45)
	Breast cancer bone metastasis	Downregulated	Bone sialoprotein, elastin, fibronectin, osteopontin, collagen I and vitronectin	Experimental (*in vitro* & *in vivo*)	Bone precursor cells, primary breast cancer mouse model	(46)
	GC	Upregulated	Immunosuppressive tumor microenvironment (TME)	Integrated bioinformatics analysis	Human gastric cancer tissue	(47, 48)
	CRPC	Upregulated	M2 tumor-associated macrophages (TAMs)	Bioinformatics analysis, *in vitro* & *in vivo* experimental	Patient samples, cell lines, Mouse xenograft models	(49, 50)
	CRC	Upregulated	TLR4	Integrated Experimental (*in vitro* & *in vivo*) & Clinical Study	Mouse models, CRC cell lines, Human CRC tissues	(51)
	Cancer Cachexia	Upregulated	IRS1	*In vivo* experimental	CTSK^+/+^ and CTSK^-/-^ mice	(52)
	OC	Upregulated	–	Experimental (*in vitro* & *in vivo*) and clinical study	OC patient tissues, OC cell lines, Mouse xenograft models	(53)
	GBM	Upregulated	Osteopontin(OPN), Chondroitin sulfate	bioinformatics Analysis and experimental validation	GBM patient tissues, GBM cell lines	(54)

CTSK is involved in the growth and metastasis of various tumors. In a cohort predominantly composed of invasive breast ductal carcinoma (n = 34), with a smaller subset of invasive breast lobular carcinoma (n = 2), Solomon et al. demonstrated that the proteolytic activities of CTSK, matrix metalloproteinase-2 (MMP-2), and MMP-9 were significantly elevated in human breast cancer tissue compared with adjacent normal tissue—suggesting a key role for these enzymes in ECM remodeling and tumor progression (45). Another study on breast cancer indicated that the Wenshen Zhuanggu formula (WSZG), composed of three medicinal herbs: *Psoraleae Fructus* (Cullen corylifolium), *Cnidii Fructus* (Cnidium monnieri), and *Aconiti Lateralis Radix Praeparata* (Aconitum carmichaelii), inhibits the formation of the pre-metastatic microenvironment by suppressing tumor-derived exosome-induced osteoclast differentiation and downregulating proteases such as CTSK, thereby maintaining ECM homeostasis (46). This is due to the fact that cell communication in breast cancer tissue is closely linked to exosomes, which play a crucial role in the interaction between tumor cells and the microenvironment (55–57). In gastric cancer (GC), a bioinformatics analysis of GC showed that high expression of CTSK in GC was significantly associated with poor prognosis. CTSK is functionally linked to ECM receptor contacts and the enrichment of the focal adhesion pathway, underscoring its pivotal involvement in ECM dynamics (47). Further studies indicate that CTSK may interact with ECM receptors via the integrin signaling pathway. Upon binding to the ECM, integrins trigger intracellular signaling cascades, such as through focal adhesion kinase (FAK) and the phosphoinositide 3-kinase/protein kinase B (PI3K/AKT) pathway (58). These pathways play crucial roles in cell proliferation, survival, migration, and invasion. Another bioinformatics study revealed that the upregulation of CTSK in GC associated with *H. pylori* infection implies that its proteolytic activity may facilitate ECM degradation and remodeling, thus advancing disease progression, suggesting that CTSK could serve as a potential diagnostic biomarker for GC (59). CTSK protein hydrolysis activity may directly promote ECM remodeling and invasive tumor behavior, and is closely related to the GC tumor microenvironment (48). Wu et al. established that in castration-resistant prostate cancer (CRPC), IL-17A-induced CTSK interferes with the E-cadherin/β-catenin complex, thereby initiating an EMT program and ECM remodeling to promote tumor metastasis (49). Another study explained another pathway: the tumor microenvironment rich in *H. pylori* can also promote CRPC metastasis through IL-17RA-mediated upregulation of CTSK (50). Collectively, these findings highlight CTSK serves as a pivotal effector in CRPC metastasis, where IL-17 signaling—triggered either directly by IL-17A or indirectly via an *H. pylori* enriched tumor microenvironment through IL-17RA. In colorectal cancer (CRC), CTSK associates gut microbiota imbalances with CRC metastasis. CTSK facilitates the establishment of an M2-type immunosuppressive microenvironment, which, in conjunction with the proteolytic activity of CTSK, expedites ECM remodeling and metastatic advancement (51). In addition to its classic role in influencing ECM dynamics, Meng et al. also revealed that CTSK drives cancer cachexia by ubiquitination and degradation of IRS1, thereby disrupting anabolic signaling and leading to severe disintegration of muscle structure and ECM homeostasis (52). During ovarian cancer (OC) pathogenesis, the suppression of EMT and metastasis by the tumor suppressor lncRNA AGAP2-AS1 is mediated through the downregulation of CTSK-a mechanism by which it ultimately curbs the protease-driven ECM remodeling essential for cancer progression (53). Unlike its established roles in other cancers, transcriptomic analysis showed that CTSK was highly overexpressed in glioblastoma multiforme (GBM), but primarily in a proenzyme form without proteolytic activity. CTSK was enriched in an inactive state, indicating that its function deviates from its classic ECM degradation role in other cancers (54). In summary, CTSK plays a central role in the progression of various solid tumors through multiple mechanisms, including regulating ECM degradation, EMT, immune microenvironment reprogramming, and activation of abnormal signaling pathways. In GBM, it exists in a non-enzymatic form, highlighting the complexity of CTSK in tumor growth and metastasis.

## 5 Relationship between cathepsin K and ECM dynamics in cardiovascular disease

Cardiovascular disease (CVD) is the predominant cause of mortality globally, responsible for over 18.5 million fatalities (9.6 million men and 8.9 million women), constituting approximately one-third of all deaths worldwide (60). CVD is the predominant cause of mortality in China, responsible for almost 4 million fatalities, underscoring its significant disease burden and increasing risk of premature death annually (61). CTSK is not only a key enzyme in bone metabolism, but it can also regulate ECM dynamics by modulating signaling pathways such as NF-κB, TLR, and RANKL/RANK/OPG, making it a potential biomarker and therapeutic target for cardiovascular diseases. Multiple studies have confirmed that CTSK may affect the development and progression of cardiovascular diseases, including atherosclerosis, heart failure (HF), ischemic cardiomyopathy, etc, by influencing ECM dynamics. **Table 3** illustrates the function of CTSK in cardiovascular disease.

**Table 3:** The function of CTSK in cardiovascular disease.

Gene	Disease	Expression	Substrates/target	Study type	Sample	References
C T S K	Doxorubicin-induced Cardiotoxicity	Upregulated	Desmin, Sarcomeric α-actinin	Experimental (*in vivo*)	Cardiomyocyte-specific CTSK knockout mouse model	(62)
	Ischemic Cardiomyopathy	Downregulated	TLR9	Experimental (*in vivo* & *in vitro*)	CTSK-deficient mice, Endothelial cells	(63)
	HF	Upregulated	–	Bioinformatics & Experimental Validation (serum of HF patients)	HF patients	(64)
	Heart Valve Development	Upregulated	Type I Collagen/Bone Matrix	Experimental (*in vivo* & ex vivo)	NFATc1^(-/-)^ mice, endocardial cushion (ECC) endothelial cells	(65)

CTSK plays multiple pathogenic and protective roles in CVD. In the pathological process of atherosclerosis and plaque formation, inflammatory factors stimulate macrophages and vascular smooth muscle cells to significantly upregulate CTSK expression. CTSK disrupts the integrity of the fibrous cap and the stability of the arterial wall by degrading collagen and elastin in the vascular wall. This promotes the formation of aneurysmal lesions and exacerbates plaque instability (66). In the same research, experiments confirmed that CTSK deficiency (CTSK^^−^/^−^^) reduced plaque formation and increased plaque fibrosis in ApoE^^−^/^−^^ mice, suggesting that CTSK promotes plaque instability and progression. In LDLR^^−^/^−^^ mice on a high-fat diet (HFD), CTSK deficiency led to reduced damage to elastic fibers and collagen, suggesting its crucial role in matrix degradation (66). Guo et al. also constructed a cardiomyocyte-specific CTSK-deficient mouse model (Myh-Cre+; Ctsk^fl/fl^) and administered doxorubicin intraperitoneally. The results showed that the cardiotoxic effects of doxorubicin were weakened or reversed in the experimental group (62). This may be related to the fact that the absence of CTSK disrupts the dynamic regulation of cardiac energy balance in the ECM, weakens the NF-κB signaling pathway, and reduces apoptosis, thus confirming the cardioprotective effect of CTSK. Conversely, in ischemic cardiomyopathy, CTSK^^−^/^−^^ mice exhibited impaired recovery of ischemic muscle function and decreased migration/invasion capacity of vascular endothelial cells (ECs) and endothelial progenitor cells (EPCs), suggesting that CTSK plays a protective role in ischemic vascular remodeling (63). However, in a bioinformatics analysis of HF, CTSK was significantly upregulated, and this was verified by *in vitro* PCR. It promotes structural damage and functional failure of myocardial tissue by mediating the abnormal degradation and remodeling of the ECM (64). In addition, CTSK is also involved in the development of heart valves, acting as a key downstream effector of the NFATc1 signaling pathway. Its expression is specifically induced by RANKL through the JNK1/2 pathway, performing ECM remodeling functions and thus promoting the transition of the endocardial cushion from the growth phase to the remodeling phase (65). In summary, CTSK plays a dual role by regulating ECM homeostasis: promoting pathological degradation of ECM leading to cardiovascular remodeling in atherosclerosis and HF, while mediating physiological remodeling of ECM to maintain tissue homeostasis in ischemic cardiomyopathy and valvular dysplasia.

## 6 Relationship between cathepsin k and ECM dynamics in pulmonary disease

Chronic respiratory diseases (CRD) have long been a global health threat, with approximately 468 million individuals suffering from the disease, 4.4 million deaths, and a heavy burden of 108.5 million disability-adjusted life years (DALYs) worldwide in 2021. Moreover, the distribution and evolution of the disease are influenced by risk factors such as the level of economic development, population aging, and external air pollution (67). Multiple studies demonstrate that CTSK-mediated ECM remodeling and EMT mechanisms are the principal factors in the development of various CRDs, such as pulmonary fibrosis (PF), chronic obstructive pulmonary disease (COPD), etc. **Table 4** delineates the role of CTSK in pulmonary disease.

**Table 4:** The function of CTSK in pulmonary disease.

Gene	Disease	Expression	Substrates/target	Study type	Sample	References
C T S K	PF	Upregulated	SNX9	Integrated Mechanistic & Clinical Study	Bleomycin (BLM)‐induced PF, Fibroblasts, PF patient serum	(68)
		Downregulated	Intracellular protein and collagen	Experimental studies (*in vitro* and *in vivo*)	Mouse lung macrophages and lung fibroblasts	(69)
	IPF	Upregulated	–	Bioinformatics & *In vivo* experimental Validation	IPF patient, Lipopolysaccharide (LPS)-induced PF	(70)
	SSc-PF	Downregulated	Collagen	Mechanistic Study, experimental studies (*in vitro*)	Primary human lung fibroblasts	(71)
	COPD	Upregulated	–	Experimental (*in vivo*) & Bioinformatics Analysis	Cigarette smoke-exposed mice, public COPD patients	(72)
		Downregulated	Intracellular protein and collagen	Experimental studies (*in vitro* and *in vivo*)	Mouse lung macrophages and lung fibroblasts	(69)
	Asthma	Upregulated	E-cadherin, Collagen I, III/EGFR	Experimental (*in vitro* and *in vivo*) & Clinical Study	house dust mite (HDM)-induced asthma model	(73)
	Tuberculosis	Upregulated	Type I Collagen, Gelatin, Type II Collagen	Experimental (*in vitro* and *in vivo*)	Rabbit cavitary lung tissue, tuberculosis patients	(74)

The pathogenesis of many CRD involves CTSK-mediated ECM remodeling and EMT mechanisms. PF is characterized by aberrant ECM remodeling, driven by excessive collagen deposition from activated fibroblasts. However, In the pathological process of PF, the role of CTSK shows bidirectional regulation. On the one hand, CTSK facilitates pathological ECM remodeling through SNX9-mediated endocytosis, activating the TGF-β1/SMAD3 signaling pathway to enhance glutaminase 1-dependent glutamine metabolism, thus promoting fibroblast collagen synthesis (68). Bioinformatics analysis using the WCGNA method also showed that CTSK, as a key node in the Idiopathic Pulmonary Fibrosis (IPF) -related protein-protein interaction network, is a key gene affecting ECM homeostasis. Predictive models showed that the survival rate of individuals with high CTSK expression was lower than that of individuals with low expression, and high expression of CTSK was observed in LPS-induced mouse models (70). On the other hand, CTSK can inhibit fibrosis. Experimental data show that lung-resident macrophages employ CTSK as a critical phagosomal collagenase to clear ECM while this homeostatic function is suppressed by profibrotic stimuli like TGF-β1, contributing to pathological ECM accumulation (69). In systemic sclerosis-associated pulmonary fibrosis (SSc-PF), the pro-fibrotic IGF-II/SOX9 axis disrupts ECM homeostasis by enhancing collagen biosynthesis and inhibiting the expression of the collagen-degrading enzyme CTSK, thereby promoting ECM accumulation. This indicates that IGF-II, by downregulating CTSK expression, weakens the lung tissue’s ability to degrade collagen, thus promoting fibrosis, and CTSK acts as an inhibitory factor in PF (71). In a study on COPD, smoke-induced upregulation of CTSK in mouse lung tissue, along with upregulation of other matrix-degrading enzymes such as MMP12, indicates that CTSK’s co-proteolytic effect on the ECM leads to abnormal ECM remodeling in COPD. Therefore, upregulation of CTSK in COPD drives the pathological progression of the disease by promoting excessive ECM degradation and alveolar structural damage (72). In the course of COPD, the proteolytic function of CTSK in pulmonary macrophage phagosomes is also impaired, which disrupts collagen clearance and links intracellular ECM degradation defects to ECM accumulation (69). Therefore, we can conclude that CTSK also exerts a bidirectional regulatory effect in COPD. In asthma, CTSK produced from airway epithelium regulates ECM remodeling by activating epithelial-mesenchymal trophic units (EMTUs) through a PAR2-mediated signaling cascade, establishing it as a fundamental factor in pathogenic airway restructuring (73). Furthermore, CTSK is a key driver of ECM dysregulation in tuberculosis, and its expression around granulomas promotes collagen dissolution and cavity formation, thus highlighting its role in immunopathology (74). Therefore, CTSK functions as a regulator of intrapulmonary ECM dynamics, possessing the dual capacity to promote pathological ECM degradation while preserving collagen homeostasis. The unique illness setting determines whether it results in ECM degradation or buildup, defining its dual role in respiratory pathology.

## 7 Relationship between cathepsin K and ECM dynamics in orthopedic diseases

Orthopedic diseases, encompassing lesions of bones, joints, muscles, ligaments, and soft tissues, are a major cause of long-term disability, particularly hip and knee arthritis and spinal disorders, which significantly limit daily activities and work capacity. With an aging population and changing lifestyles, the incidence and burden of orthopedic diseases are on the rise. CTSK significantly influences the progression of orthopedic diseases by regulating the degradation and remodeling of the ECM (2, 75). **Table 5** delineates the role of CTSK in orthopedic diseases.

**Table 5:** The function of CTSK in orthopedic diseases.

Gene	Disease	Expression	Substrates/target	Study type	Sample	References
C T S K	OA	Upregulated	Collagen	Database Analysis (GWAS Analysis)	OA patients	(76)
	OA	Upregulated	Subchondral bone	Clinical studies & *In vitro* and *in vivo* experimental studies	OA patients, destabilization of the medial meniscus (DMM)-induced murine OA model, osteoclast differentiation system	(77)
	Age-dependent Osteoarthritis	Upregulated	–	Experimental (*in vivo*)	αCGRP-deficient mice	(78)
	Glucocorticoid-induced OP	Upregulated	Type I collagen	*In vitro* cell study	MLO-Y4 Osteoblast-Like Cell Line	(79)
	OP	Upregulated	Type I Collagen	Experimental (*in vitro* & *in vivo*)	bone marrow macrophages (BMMs), RAW264.7 cells, ovariectomized (OVX) mice model	(80)
	Postmenopausal OP (PMOP)	Upregulated	–	Integrative Multi-omics & Computational Study	OVX-induced PMOP rat model, Fecal samples, Computational models	(81)
	Type 2 Diabetes Osteoporosis (T2DOP)	Upregulated	–	Experimental (*in vivo*)	T2DOP model in db/db mice	(82)

CTSK orchestrates the pathogenesis of osteoarthritis (OA) and osteoporosis (OP) by dynamically remodeling the ECM, a central mechanistic insight that will be further dissected in the following section. The investigation of OA reveals that the GWAS-identified risk gene CTSK directly cleaves type I and II collagen in the articular cartilage ECM, this abnormal proteolytic activity disrupts ECM homeostasis, leading to cartilage degradation and contributing to the pathogenesis of osteoarthritis (76). CTSK also participates in the reprogramming mechanism. The reprogramming mechanism involving CTSK is significantly potentiated post-activation, where it executes its function by degrading the ECM; this catabolic action directly mediates subchondral bone resorption and microarchitectural disruption (77). Alexander et al. used αCGRP knockout mice (CGRP^-/-aged^) and assessed OA progression using OARSI pathological grading and μCT scans (78). Their results showed that age-related OA in WT_aged_ mice was accompanied by upregulated CTSK expression, while this phenomenon was not observed in αCGRP^-/-aged^ mice. This further demonstrates that CTSK plays a key role in the imbalance between cartilage degeneration and bone remodeling in OA by regulating the dynamic balance of the ECM through its proteolytic activity. In OP, CTSK serves as a convergent downstream effector that integrates diverse pathological signals to drive bone loss across multiple forms of OP, primarily through its collagenolytic degradation of the ECM. Yuan et al. demonstrated that glucocorticoid stress induces a dysregulated PINK1-mediated mitophagy in osteocytes, leading to excessive CTSK production, which subsequently degrades the adjacent type I collagen network, revealing a new mechanism of osteocyte-mediated peri-lacunar/canalicular ECM degradation in glucocorticoid-induced OP (79). Another study demonstrated that Urolithin B attenuates osteoporotic bone loss by suppressing osteoclastogenesis and the expression of CTSK via downregulating the ERK/NF-κB signaling pathway, thereby preserving ECM integrity (80). Research on postmenopausal OP (PMOP) indicates that Roucongrong Pill mitigates PMOP by regulating gut microbiota and host metabolites, consequently decreasing critical variables such as CTSK activity, which reduces ECM disintegration and bone loss (81). Similarly, in the context of Type 2 Diabetes Mellitus (T2DM) -related OP, Gegen Qinlian Decoction (GQD), composed of *Pueraria lobata*, *Scutellaria baicalensis*, *Coptis chinensis*, and *Glycyrrhiza uralensis*, mitigates bone loss by downregulating the expression of CTSK via suppressing the IGFBP3/MAPK/NFATc1 signaling axis, thereby preserving ECM integrity (82). CTSK is clearly pivotal in the ECM dynamics in OP, irrespective of upstream mechanism. Therefore, across diverse orthopedic diseases, CTSK emerges as a pivotal downstream effector which integrates disparate upstream signals to catalyze the degradation of collagenous components within the ECM, thereby fundamentally driving the aberrant ECM dynamics to OA and OP progression.

## 8 Relationship between cathepsin K and ECM dynamics in metabolic diseases

Metabolic diseases, including diabetes and obesity, are rapidly increasing globally, imposing a heavy burden on human health and socioeconomic status. Based on data from the Global Burden of Disease Study 2021, the burden of metabolic diseases is substantial, with type 2 diabetes (T2DM) affecting approximately 510 million people in 2021 and its prevalence continuing to rise. The trend in DALYs for T2DM has also increased, underscoring the urgent need for enhanced public health responses (83). Meanwhile, obesity (BMI ≥ 30 kg/m²) affects more than 10% of the adult population, with an average annual growth rate of approximately 0.70% (83). Diabetes and obesity are mutually influential; obesity is a major risk factor for T2DM, while high BMI-induced diabetes DALYs increase by an average of 1.82% annually (84). These two metabolic diseases together lead to cardiovascular, renal, and retinal complications, significantly increasing all-cause mortality and healthcare expenditure, and have become core drivers of the burden of non-communicable diseases (NCDs) (85). Research indicates that CTSK plays a crucial role in tissue remodeling and pathological processes in metabolic diseases by degrading and remodeling the ECM (2). **Table 6** delineates the role of CTSK in metabolic diseases.

**Table 6:** The function of CTSK in orthopedic diseases.

Gene	Disease	Expression	Substrates/target	Study type	Sample	References
C T S K	T2DM-associated Bone Fragility	Upregulated	Bone Matrix, Extracellular Matrix	Experimental (*in vivo* & *in vitro*)	T2DM db/db mice models	(86)
	Diabetic Wound Healing	Upregulated	Collagen and elastin	Experimental Study (*in vivo* & *in vitro*)	Diabetic pigs (STZ-induced), CTSK-/- diabetic mice, Fibroblasts, Keratinocytes	(87)
	Obesity	Upregulated	–	Experimental (*in vivo*)	D-galactose-induced Wistar rats	(88)
	T2DM/High-Fat Diet (HFD)-induced Bone Loss	Upregulated	–	Experimental (*in vivo*)	T2DM mice (HFD+STZ), Adipose-specific chemerin knockout mice	(89)
	Diet-induced bone metabolic imbalance.	Upregulated/downregulated	–	Experimental (*in vivo*)	HFD mice and high-fructose diet (HFrD) mice	(90)

CTSK contributes to T2DM and obesity by disrupting ECM dynamics and promoting metabolic disorders such as insulin resistance. Wu et al. found that CTSK is upregulated by locally accumulated sclerostin in the osteocyte lacunar-canalicular system (LCS), serving as a critical executor of pathologic ECM degradation that compromises bone quality in T2DM mice (86). Conversely, blocking CTSK can reduce pathological ECM breakdown in diabetic wounds, hence improving ECM stability and facilitating wound healing (87). In the process of obesity, Napatsorn et al. demonstrated through transcriptional analysis of an obese aged rat model that hyperbaric oxygen therapy (HBOT) can inhibit CTSK expression, which is a key mechanism by which it limits the hydrolytic destruction of bone ECM and improves osteoporosis (88). A HFD promotes obesity, which is one of the primary risk factors for the onset of T2DM. Both of these metabolic disorders are associated with CTSK-induced ECM remodeling. In metabolic diseases (T2DM and obesity), reduced chemerin enhances bone anabolism and suppresses osteoclast activity, the latter evidenced by decreased CTSK expression, thereby attenuating pathological degradation of the bone ECM and contributing to exercise-induced bone improvement (89). Extending beyond these established metabolic disorders, Tian et al. demonstrate that in nutritional metabolic perturbations, CTSK dynamically driving the distinct bone mass trajectories observed under high-fat and high-fructose diets (90). CTSK expression is upregulated early in dietary intervention, particularly during prolonged bone loss in high-fat diets, suggesting it mediates diet-induced bone metabolic imbalance (90). In summary, CTSK functions as a core hub that integrates diverse pathological signals (e.g., sclerostin, chemerin, hyperglycemia) in T2DM and obesity, executing their biological effects through the degradation of the ECM in bone and skin.

## 9 Potential risks of targeted CTSK therapy

One primary concern regarding CTSK inhibitors is the potential for off-target effects and selectivity issues. CTSK is a member of the cysteine protease family, and its active site shares homology with other cysteine proteases, such as CTSS (91). This structural similarity can lead to non-selective inhibition, potentially affecting the normal physiological functions of other cathepsins and resulting in unintended side effects (91). Because of its expression in immune cells such as dendritic cells, macrophages, and T cells beyond osteoclasts, non-selective inhibition of CTSK raises concerns about impairing the proper functioning of these cells and other systems where it is active (91, 92). Clinical experience and animal model studies have indicated other potential side effects associated with CTSK inhibitors. These include mild elevations in liver enzymes, arthralgia (joint pain), and rare photosensitivity reactions (93). CTSK has also undertaken rigorous and clinically meaningful trials. Although MIV-711, a CTSK inhibitor, demonstrated a favorable safety and tolerability profile in a 26-week Phase IIa clinical trial, with the most common adverse events being nasopharyngitis, arthralgia, and headache, its long-term safety requires further assessment (93). Moreover, studies indicate that CTSK deficiency may aggravate the hemorrhagic transformation caused by recombinant tissue plasminogen activator (rt-PA) during ischemic stroke and may possibly affect thrombus formation (91). This indicates a complex role for CTSK in the cardiovascular system that necessitates a deeper understanding to fully characterize the risk profile of its inhibitors (91). The observation that odanacatib, after prolonged use (≥5 years) in the LOFT Phase III clinical trial, was associated with a small but statistically significant increased risk of stroke, significantly impacted the development landscape for CTSK inhibitors (91).

Given these concerns, current research efforts are focused on developing next-generation CTSK inhibitors with enhanced selectivity and reduced central nervous system (CNS) penetration to mitigate the risk of stroke (91, 93). Researchers are investigating alternative inhibition strategies, such as allosteric (ectosteric) inhibition, which targets regulatory sites on CTSK distinct from the catalytic active site (94). Sophoraflavanone G (SG), a naturally derived ectosteric CTSK inhibitor, has demonstrated efficacy in attenuating ovariectomy-induced bone loss in preclinical models by selectively suppressing osteoclastic bone resorption—suggesting a potentially improved safety profile relative to conventional active-site inhibitors, which may be associated with off-target effects (94).

Although no CTSK inhibitors have yet received regulatory approval for widespread clinical use, the development of new, highly selective inhibitors like MIV-711 and H-9 is ongoing (93, 94). These efforts aim to optimize the therapeutic window—balancing efficacy against safety—by leveraging the well-established, multifaceted role of CTSK in diverse pathological processes, including bone disorders, cancer, and inflammatory conditions (4, 92).

## 10 Conclusion

In summary, this review establishes CTSK as a key regulator influencing ECM dynamics, with functions extending beyond its classic role in bone resorption. Under various pathological conditions, including malignancies, cardiovascular and pulmonary diseases, orthopedic diseases, and metabolic disorders, CTSK acts as an executor of ECM degradation and remodeling, and its activity is regulated by a complex upstream signaling network. Dysregulation of CTSK disrupts tissue homeostasis, thereby promoting tumor invasion and metastasis, driving cardiovascular disorder and pulmonary fibrosis, exacerbating musculoskeletal degeneration, and impairing tissue repair function in metabolic diseases such as T2DM and obesity.

Future research should focus on the biological role of CTSK, emphasizing the development of highly selective targeted drugs to regulate CTSK activity in specific tissues and pathophysiological environments. Translating this basic research into effective clinical interventions is crucial, ultimately opening up new treatment options for diseases involving ECM dynamics.

## Glossary

BMI: Body Mass Index
CNS: Central Nervous System
COPD: Chronic Obstructive Pulmonary Disease
CRC: Colorectal Cancer
CRD: Chronic Respiratory Diseases
CRPC: Castration-Resistant Prostate Cancer
CTSK: Cathepsin K
CTSL: Cathepsin L
CTSS: Cathepsin S
CVD: Cardiovascular Disease
DALYs: Disability-Adjusted Life Years
ECs: Endothelial Cells
ECM: Extracellular Matrix
EMT: Epithelial-Mesenchymal Transition
EMTUs: Epithelial-Mesenchymal Trophic Units
EPCs: Endothelial Progenitor Cells
ERK: Extracellular Signal-Regulated Kinase
FAK: Focal Adhesion Kinase
GAGs: Glycosaminoglycans
GBM: Glioblastoma Multiforme
GC: Gastric Cancer
GQD: Gegen Qinlian Decoction
GWAS: Genome-Wide Association Study
HBOT: Hyperbaric Oxygen Therapy
HF: Heart Failure
HFD: High-Fat Diet
HFrD: High-Fructose Diet
IGFBP3: Insulin-like Growth Factor Binding Protein 3
IPF: Idiopathic Pulmonary Fibrosis
IRS1: Insulin Receptor Substrate 1
JNK: c-Jun N-terminal Kinase
LCS: Lacunar-Canalicular System
MAPK: Mitogen-Activated Protein Kinase
MMP: Matrix Metalloproteinase
NCDs: Non-Communicable Diseases
NFATc1: Nuclear Factor of Activated T cells, cytoplasmic 1
NF-κB: Nuclear Factor kappa-B
OA: Osteoarthritis
OC: Ovarian Cancer
OPG: Osteoprotegerin
PF: Pulmonary Fibrosis
PI3K: Phosphoinositide 3-kinase
PMOP: Postmenopausal Osteoporosis
RANK: Receptor Activator of Nuclear Factor Kappa-B
RANKL: Receptor Activator of Nuclear Factor Kappa-B Ligand
SNX9: Sorting Nexin 9
SSc-PF: Systemic Sclerosis-associated Pulmonary Fibrosis
T2DM: Type 2 Diabetes Mellitus
T2DOP: Type 2 Diabetes Osteoporosis
TGF-β1: Transforming Growth Factor-beta 1
TLR: Toll-Like Receptor
TME: Tumor Microenvironment
TNF-α: Tumor Necrosis Factor-alpha
TRAF6: Tumor Necrosis Factor Receptor-Associated Factor 6
WSZG: Wenshen Zhuanggu Formula
